# High-quality genome assembly of a cosmopolitan insect predator, *Chrysoperla zastrowi sillemi* (Esben-Petersen)

**DOI:** 10.1038/s41597-025-04571-2

**Published:** 2025-02-16

**Authors:** Muthugounder Mohan, Thiruvengadam Venkatesan, Selvapandian Upasna, Selvamani Selva Babu, P J Aneesha, Karuppannasamy Ashok, Budhwar Roli, Gandhi R Gracy, Satya N Sushil

**Affiliations:** 1 Division of Genomic Resources, ICAR- National Bureau of Agricultural Insect Resources, Hebbal, Bengaluru, 560024 India; 2 Tata Institute for Genetics and Society, Bengaluru, 560065 India; 3 Bionivid Technology Private Limited, Bengaluru, 560043 India

**Keywords:** Entomology, Genome evolution

## Abstract

*Chrysoperla zastrowi sillemi* is an extensively employed bio-control agent against crop pests. However, the genomic information on adaptive evolution and predator-prey interaction was lacking to enhance its predatory potential. Here, we presented a high contiguity, chromosomal-level genome assembly of *C. zastrowi sillemi* using short and long-read sequencing (Illumina and PacBio) coupled with Hi-C. The highly homozygous genome assembly has 597 Mb in span. The assembly contains six chromosomal pseudomolecules, occupying 84% of genome, which range from 29.36 to 170.05 Mb. Genome annotation using Refseq, EggNOG, SwissProt and Eukaryotic Orthologous Groups database has identified 14,495 protein coding genes. The mitochondrial genome is 16 Kb in length with 13 protein-coding genes, 22 tRNAs and three rRNA genes without any gene rearrangements. The genomic information furnished herein could be useful in choosing heritable traits for selective breeding to improve its bio-control potential.

## Background & Summary

An essential component of integrated pest management is biological control which uses natural enemies to control insect pests^1^. *Chrysoperla zastrowi sillemi* (Esben-Petersen) (Neuroptera: Chrysopidae), commonly known as the green lacewing or aphid lion is a potential predator that is a key component of biological control in many crop ecosystems^2,3^. It has high predatory potential, and feeds mainly upon mites, aphids, coccids, leafhoppers and small lepidopteran larvae^4^. Many studies have proved the predatory efficacy of *C. zastrowi sillemi* under laboratory and field conditions^5,6^. For unravelling the functional genomics, it is crucial to obtain the whole-genome information of the predatory species.

**Fig. 1:**
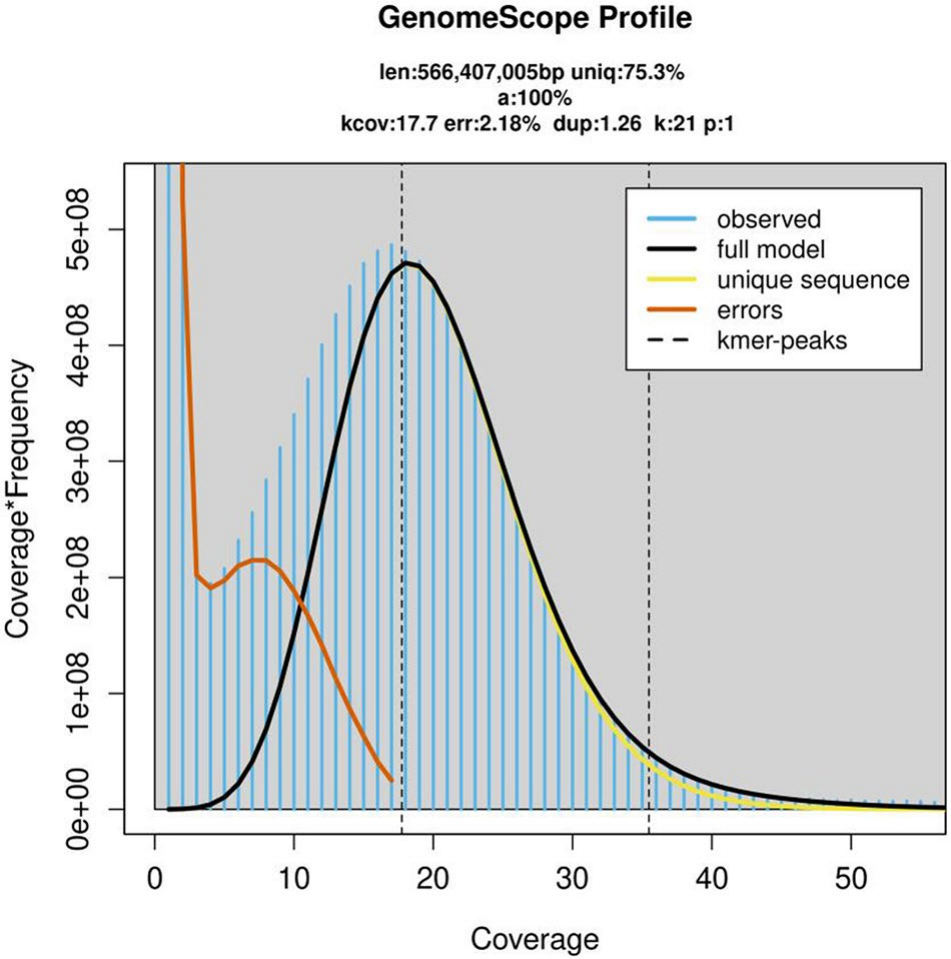
K-mer frequency distribution plotted by GenomeScope from Illumina short reads for the estimation of genome size of *C. zastrowi sillemi*. The distribution was determined with jellyfish using a k-mer size of 21. The highly homozygous nature of the genome is visualized by the absence of k-mers in the heterozygous region.

In the present study, the genome of *C. zastrowi sillemi* was sequenced using PacBio long read and Illumina paired-end short read sequencing. In addition, transcriptome sequencing was done to predict genes with more accuracy. The k-mer analysis estimated its genome size as 566 Mb. The k-mer distribution curve indicates the genome of the highly homozygous in nature (Fig. 1).

**Fig. 2:**
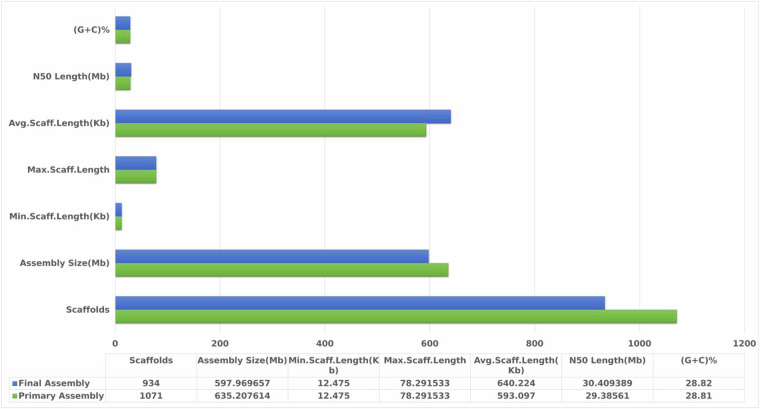
Scaffold level genome assembly statistics of *C. zastrowi sillemi*.

The raw sequence reads yielded 15 Gb of filtered PacBio HiFi reads with an average read length of 12,416 bp. Hifiasm assembler was used to create haplotype resolved assembly using HiFi and Hi-C data. The assembled genome of *C. zastrowi sillemi* has 597 Mb with scaffold N50 of 30.4 Mb (Fig. 2). The genome was scaffolded into six chromosomal pseudomolecules covering 84% of the genome. The size of the pseudochromosomes ranged from 29.37 Mb to 170.05 Mb with total size of 502 Mb (Figure S1). In Hi-C interaction, a broader view of interactions across the entire chromosome or genome is identified as linear (Figure S2a), which means the a strong interaction signal observed between two genomic regions that are located relatively close to each other on the linear chromosome^7^.The higher cis/trans ratio indicates enrichment for within the chromosomal reads (Figure S2b). The cis long/short ratio of >1.5 also suggested that the Hi-C experiment captured long - distance chromatin interactions. The summary statistics of Hi-C data is presented in Figure S3.

**Fig. 3:**
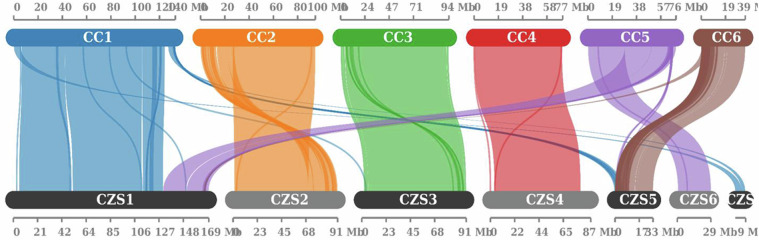
Synteny blocks display high degree of conservation of regions of chromosomes between the genomes of *C. carnea* (CC) and *C. zastrowi sillemi* (CZS).

The synteny analysis showed a strong collinearity between the *C. zastrowi sillemi* and *Chrysoperla carnea* genomes (Fig. 3). A total of 14,495 protein-coding genes, after removing the repeat sequences, were functionally annotated using Refseq, EggNOG, SwissProt and Eukaryotic Orthologous Groups (KOG) databases (Table S1).

Further, 4,572 non-coding RNAs were annotated and classified into 1,175 rRNAs, 54 miRNAs, 47 snRNAs, 125 spliceosomal RNAs and 2,070 tRNAs. The 342.5 Mb repeat elements constitute 57.31% of the genome, in which a high proportion of unclassified repeats (53.8%) were recognized. The unclassified repeats are a common phenomenon in early diverged insect orders such as Neuroptera (Table S2). The complete mitochondrial genome of *C. zastrowi sillemi* was predicted to be 16,048 bp with GC content of 21.1% containing 22 tRNA genes, 13 protein-coding genes and 3 rRNA genes (Figure S4 and Table S3).

The genome information provides a crucial resource in choosing heritable traits for a selective breeding programme. It also helps in further in-depth understanding of various aspects of its life history traits including the evolution of predator-prey interaction and its ecological adaptation.

## Materials and Methods

### Insect culture

A laboratory reared population of *C. zastrowi sillemi* (National Accession No. NBAII-MP- TRI-15) is being maintained at ICAR-National Bureau of Agricultural Insect Resources (NBAIR), Bengaluru for more than 100 generations^8^. Freshly emerged and starved adults were used for genome sequencing. DNA and RNA Extraction DNA from both male and female adults was isolated using the DNAesay® blood and tissue kit (Qiagen) following the manufacturer’s protocol. The insects were starved for 24 hours before DNA isolation. Total RNA was isolated from adult, larva and pupae of *C. zastrowi sillemi* using an RNAeasy® kit (Qiagen) following the manufacturers’ protocol.

### Genome sequencing

For the primary assembly, the DNA library was prepared with the SMRTbell® express template prep kit 2.0 to generate Hifi reads on the PacBio Sequel II platform. In addition, short-read deep sequencing was done by generating high-quality RNA and DNA libraries following the manufacturer’s protocols of NEBNext’s Ultra II FS DNA Library Prep Kit and Ultra™ II Directional RNA Library Prep Kit for Illumina®, respectively for assembly polishing and validation. Hi-C sequencing was done to detect the chromatin structures and interactions in the nucleus. Arima Genome-Wide HiC+ kit was used to prepare a high-quality library and sequenced with paired-end chemistry on Illumina Novaseq6000. Quality-based filtering (phred score threshold of 30) and adapter trimming were performed using fastp. Transcriptome sequencing was done for enhanced gene prediction on a flow cell using the Illumina NovaSeq6000 platform. The genome size was estimated using Jellyfish^9^, a k-mer based counter. GenomeScope2 was used to analyze the Jellyfish results to obtain information such as heterozygosity, repeat length and genome size.

### Genome assembly

PacBio HiFi reads were directly used to generate the primary assembly. The Hifiasm^10^ assembler was used in the Hifi-only mode to obtain a haplotype-resolved assembly. A transcriptome assembly from the RNA-seq data was generated using the Trinity ^11^ de novo assembler. CDHIT-EST^12^ was used to cluster together similar nucleotide sequences within the assembly based on the sequence and length similarity. Transcripts that were 90% identical in length and sequence similarity were grouped together and only a representative transcript (Unigene) was retained.

### Hi-C scaffolding and assembly polishing

The HiCUP^13^ pipeline was used to align the reads against the assembly and filter out the artificial experimental artefacts. Further, this alignment information was used by the YAHS^14^ pipeline to join the gaps, re-orient sequences and produce more contiguous scaffolds/pseudo- molecules. The assembly was screened for possible bacterial contamination using Kraken2^15^. The standard database containing archaea, bacteria, viral, plasmid, human and UniVec Core sequences was used to screen the assembly.

### Assembly validation

The whole genome and RNA-seq data were mapped back to the assembly with BWA^16^ and Hisat2^17^ aligners as a means of validation. Benchmarking Universal Single-Copy Orthologs (BUSCO)^18^ was used as a qualitative measure to ensure assembly completeness and annotation. The insecta_db10 database was used to assess the completeness of the *C. zastrowi sillemi* assembly. Similarly, Core Eukaryotic Genes Mapping Approach (CEGMA)^19^ that works with a database of 248 conserved eukaryotic genes was used to assess assembly completeness and annotation using Hidden Markov Models (HMMs).

### Repeat prediction

Repeat sequences were identified in the assembly using RepeatMasker^20^ that was used to soft mask the repeat regions identified with the Dfam (v3.6)^21^ database and a custom library was createdusingpredictionsfromRepeatModeller^22^, Transposon-psi^23^ and LTRharvest^24^. The custom FASTA sequences were clustered together using USEARCH to remove redundant sequences and provided to RepeatMasker as a reference for the second round of repeat masking.

### Gene prediction

The Maker2 pipeline was utilized for gene prediction. It is a wrapper package that encompasses the ab initio gene predictors Augustus^25^ and GeneMark-ES and correlates transcript and protein evidence from related species to predict genes in the assembly. The Prothint^26^ pipeline that uses the arthropoda_odb10 dataset from OrthoDB was used to score hints in the form of splice sites, introns, start and stop codons from the assembly. GeneMark EP + and the protein hints obtained from the orthologous protein sequences were used for accurate gene predictions. Chrysopidae family proteins were downloaded from the NCBI database and the de novo assembled transcriptome assembly was used as evidence for the gene prediction in Maker2^27^. An Annotation Edit Distance (AED) value of less than one and sufficient exons and splice site coverage by alignment evidence were kept as parameters for filtering the predicted gene.

### Genome annotation

Protein sequences from NCBI Refseq database were used to annotate the predicted transcripts/proteins. NCBI Blast + (v2.11)^28^ was used for the annotation. The proteins were further classified into functional annotation categories such as Gene Ontology, eukaryotic orthologous groups (KOGs), KEGG pathways and PFAM domains using the EggNOG mapper^29^ based on the EggNOG database^29^.

### Miscellaneous chromosomes

The X chromosome was identified from the synteny analysis with the published *C. carnea* X chromosome (NCBI accession: NC058342.1). The synteny was generated with the D- GENIES^30^ software which computes large genome alignments with the minimap2 aligner. In addition, the mitochondrial assembly was identified using the assembly coverage information and homology alignment with other published assemblies of the Chrysopidae family. Mitochondrial genes were predicted using the Mitos2^31^ web server.

### Synteny analysis

MCScanX^32^ which uses the MCScan algorithm was used to compute the syntenic blocks and co-linear segments among chromosomes. The *C. zastrowi sillemi* genome assembly was compared with *C. carnea* genome assembly was compared with and the synteny was viewed on the SynVisio online portal (https://synvisio.github.io/)^33^.

## Data Records

This Whole Genome Shotgun project has been deposited at NCBI GenBank under the accession JBFCZE000000000.1^34^ and GenBank assembly accession GCA_040669965.1. All the raw data files used in genome assembly such as PacBio, Illumina, and Hi-C sequencing data were deposited at the Sequence Read Archive (SRA) at NCBI with the accession number SRP410139^35^ under BioProject accession number PRJNA905226. The Annotation file published in Figshare (10.6084/m9.figshare.28099868)^36^.

## Technical Validation

Validations were employed to assess the contiguity, accuracy and completeness of the genome assembly. The final assembly validation resulted in the mapping of 85.76% and 95.50% of whole genome sequencing and Illumina raw data, respectively (Figure S5). The BUSCO analysis showed a high degree of completeness (99.0% and 94.3%) when compared with the Arthropoda data set of the Benchmark of Universal Single-Copy Orthologs (BUSCO) and Core Eukaryotic Genes Mapping Approach (CEGMA) using the eukaryotic data sets. This also included 95.3% complete and single-copy BUSCO genes and 3.7% duplicated BUSCOs. NCBI RefSeq database records matched with 80.42% of the annotated genes, and 76.28%, 67.05% and 49.79% of the genes matched with EggNOG, SwissProt and KOG databases, respectively (Table S2).

## Supplementary information

Supplementary table

Supplementary figure

## Data Availability

The present study did not use any custom scripts. All the data processing was done using the guidelines of standard pipelines and bioinformatics tools given in the methods section. Parameters for each software were detailed.
