# Supplementary material for: High-quality genome assembly of a cosmopolitan insect predator, Chrysoperla zastrowi sillemi (Esben-Petersen)
Source: Sci Data. 2025 Feb 16;12:281. doi: 10.1038/s41597-025-04571-2 (PMC11830793; doi:10.1038/s41597-025-04571-2)
Supplement: Supplementary file 1 — Supplementary table [file 41597_2025_4571_MOESM1_ESM.pdf]

High-quality genome assembly of a cosmopolitan predator, *Chrysoperla zastrowi sillemi* (Esben - Petersen)

Muthugounder Mohan[1]*, Thiruvengadam Venkatesan[1], Selvapandian Upasna[1], Selvamani Selva Babu[1], Aneesha P J[1], Karuppannasamy Ashok[1,2], Roli Budhwar[3], Gandhi R. Gracy[1], Satya N. Sushil[1]

[1]Division of Genomic Resources, ICAR- National Bureau of Agricultural Insect Resources, Hebbal, Bengaluru - 560024, Karnataka, India.

[2]Tata Institute for Genetics and Society, Bengaluru - 560065, Karnataka, India.

[3]Bionivid Technology Private Limited, Banaswadi, Bengaluru - 560043, Karnataka, India.

***Correspondence:** Muthugounder Mohan (Mohan.M@icar.gov.in)

Table S1. Summary of protein-coding genes in the genome of *C. zastrowi sillemi*

Database	Annotated number	Percentage (%)
NCBI Refseq	11657	80.42
EggNOG	11057	76.28
SWISS-Prot	9719	67.05
KOG (Eukaryotic Orthologous Group)	7217	49.79

Table S2. The distribution of major repeat elements in *C. zastrowi sillemi*

Components	Size in (Mb)	No. of Repeats
Retroelements	46.523981	283,149
LTR elements	11.472264	25,680
Transposons	33.224831	248,499
Rolling circles	65.796952	518,423
Unclassified	184.3636731	847,089
Small RNA	5.64294	34,548
Satellites	1.054608	5,816
Simple Repeat	7.016006	154,691

Low complexity	1.476871	30,533

Table S3. Summary of the mitogenome of *C. zastrowi sillemi*

Gene	Location	Strand	Size
trnV	592-662	-	71
trnI	2673-2737	+	65
trnQ	2812-2880	-	69
trnM	2883-2950	+	68
trnC	3963-4025	-	63
trnW	4026-4092	+	67
trnY	4095-4162	-	68
Cox1	4176-5684	+	1509
trnL2	5704-5768	+	65
Cox2	5776-6444	+	669
trnK	6461-6531	+	71
trnD	6532-6596	+	65
Atp8	6597-6752	+	156
Atp6	6749-7417	+	669
Cox3	7426-8211	+	786
trnG	8214-8278	+	65
nad3	8297-8623	+	327
trnA	8631-8696	+	66
trnR	8698-8761	+	64
trnN	8761-8827	+	67
trnS1	8828-8894	+	67
trnE	8898-8964	+	67
trnF	8963-9028	-	66
nad5	9033-10733	-	1701
trnH	10755-10818	-	64
trnT	12448-12511	+	64
trnP	12512-12577	-	66
cob	13106-14182	+	177
