# Supplementary material for: High-quality genome assembly of a cosmopolitan insect predator, Chrysoperla zastrowi sillemi (Esben-Petersen)
Source: Sci Data. 2025 Feb 16;12:281. doi: 10.1038/s41597-025-04571-2 (PMC11830793; doi:10.1038/s41597-025-04571-2)
Supplement: Supplementary file 2 — Supplementary figure [file 41597_2025_4571_MOESM2_ESM.pdf]

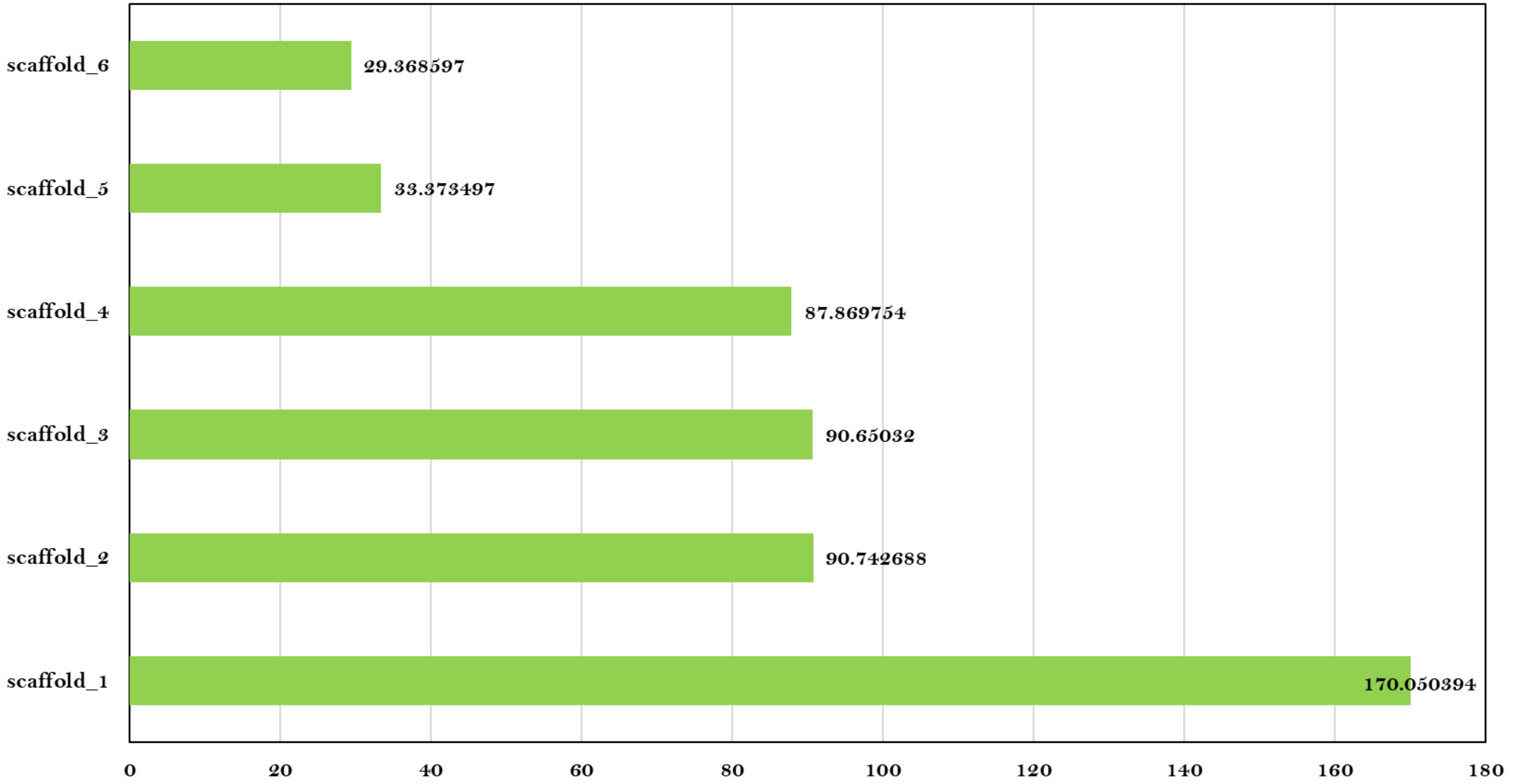

Figure S1. Length distribution of chromosomal pseudomolecules in *C. zastrowi sillemi* genome assembly

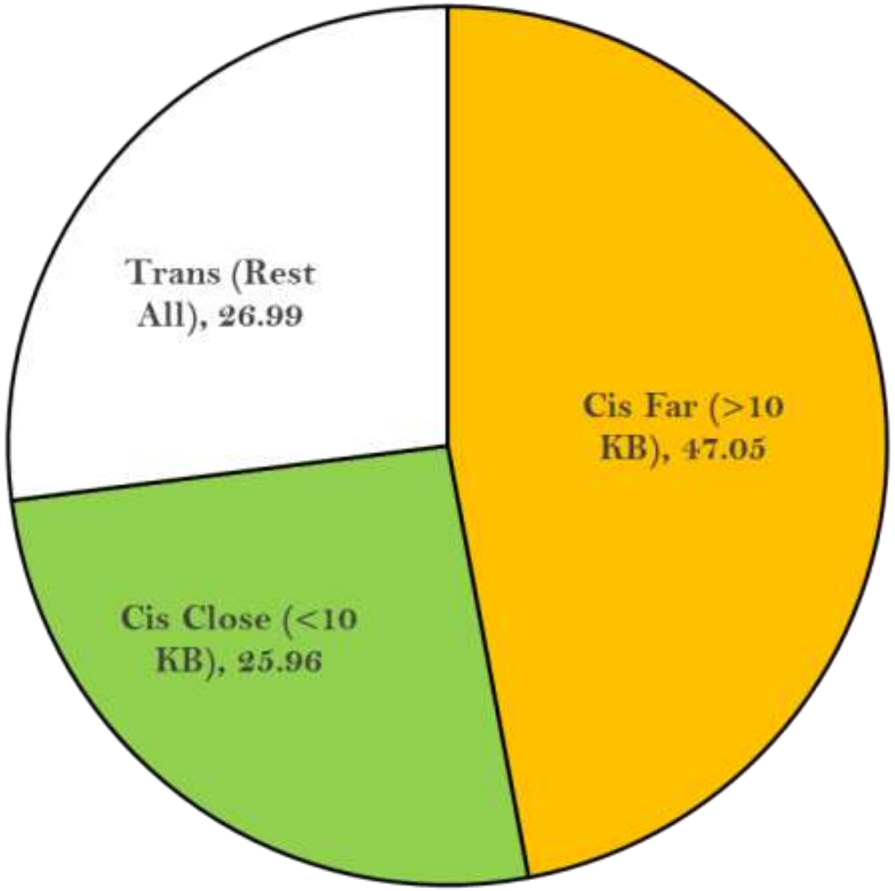

Figure S2. Hi-C data statistics (Cis/Trans ratio) of *C. zastrowi sillemi*. The higher cis/trans ratio indicates enrichment for within-chromosomal reads, expected in the Hi-C experiments

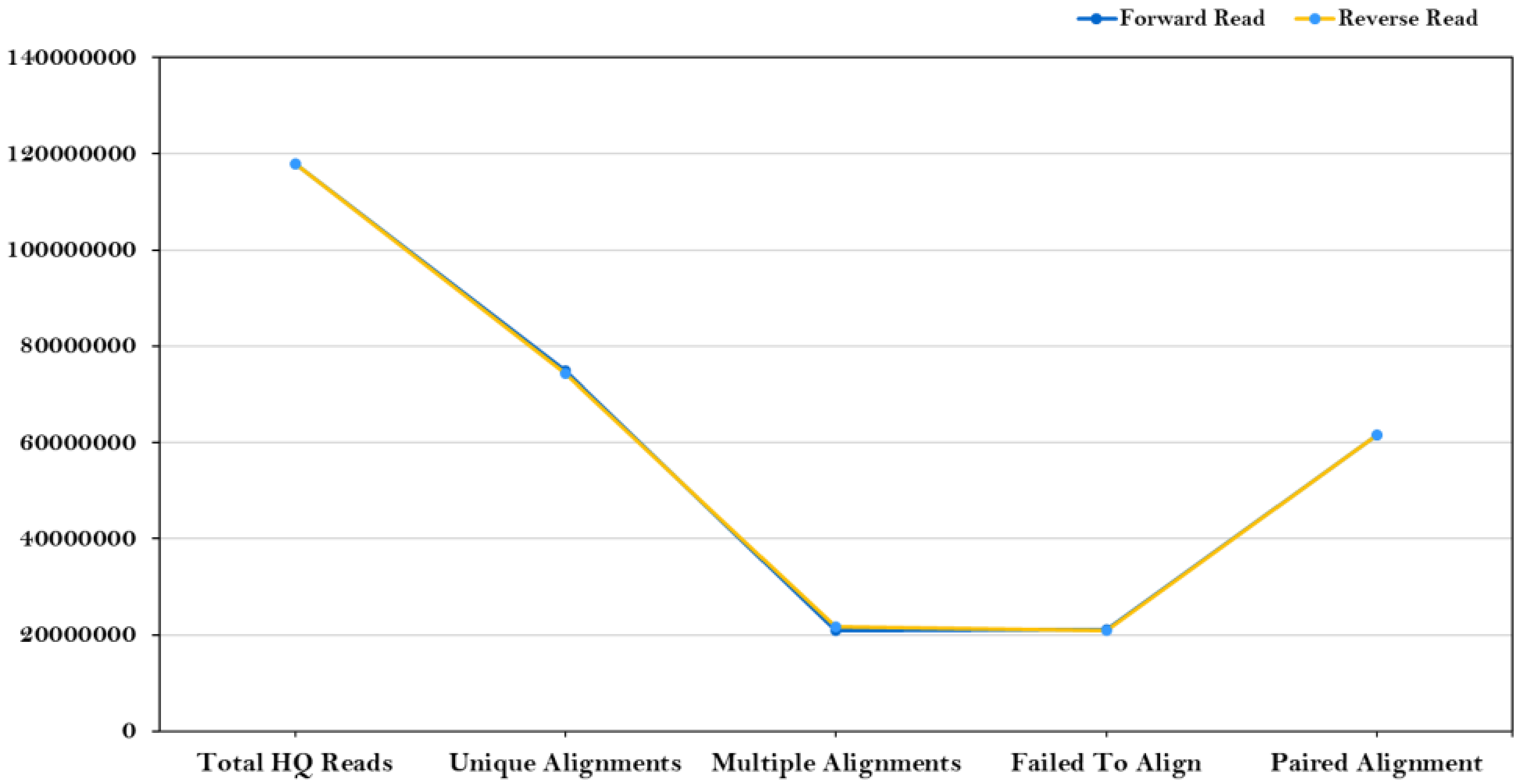

Figure S3. Summary statistics of Hi-C data of *C. zastrowi sillemi*. The library was prepared using the Arima Hi-C Kit. A total of ~36 GB data was generated using a 2x150 bp module.

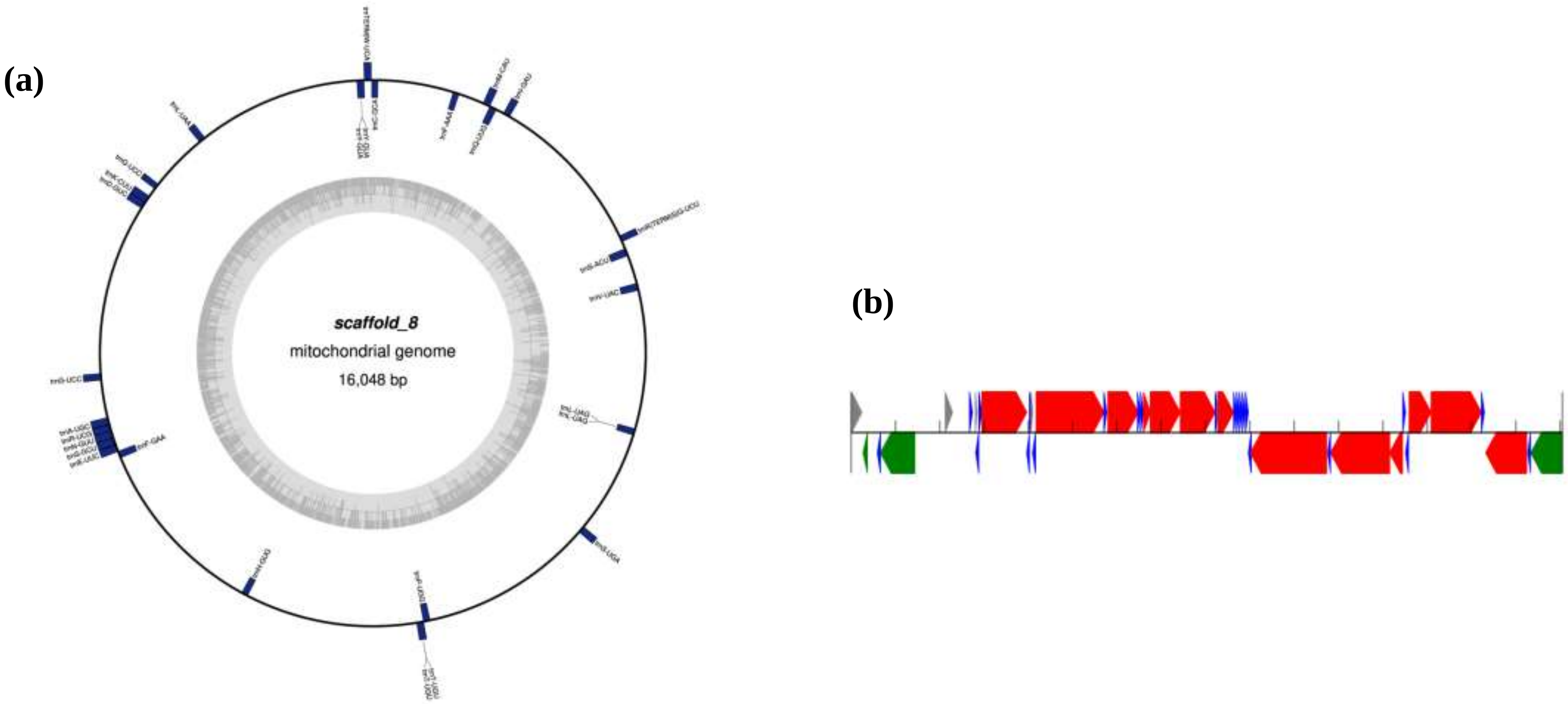

Figure S4. The mitochondrial genome of *C. zastrowi sillemi* (a) Distribution of transfer RNAs (tRNAs) (in blue) in the mitochondrial genome (b) Position of tRNA genes, protein-coding genes and rRNA genes. Blue, red and green color represents the tRNA genes (22), protein-coding genes (13) and rRNA genes (3)

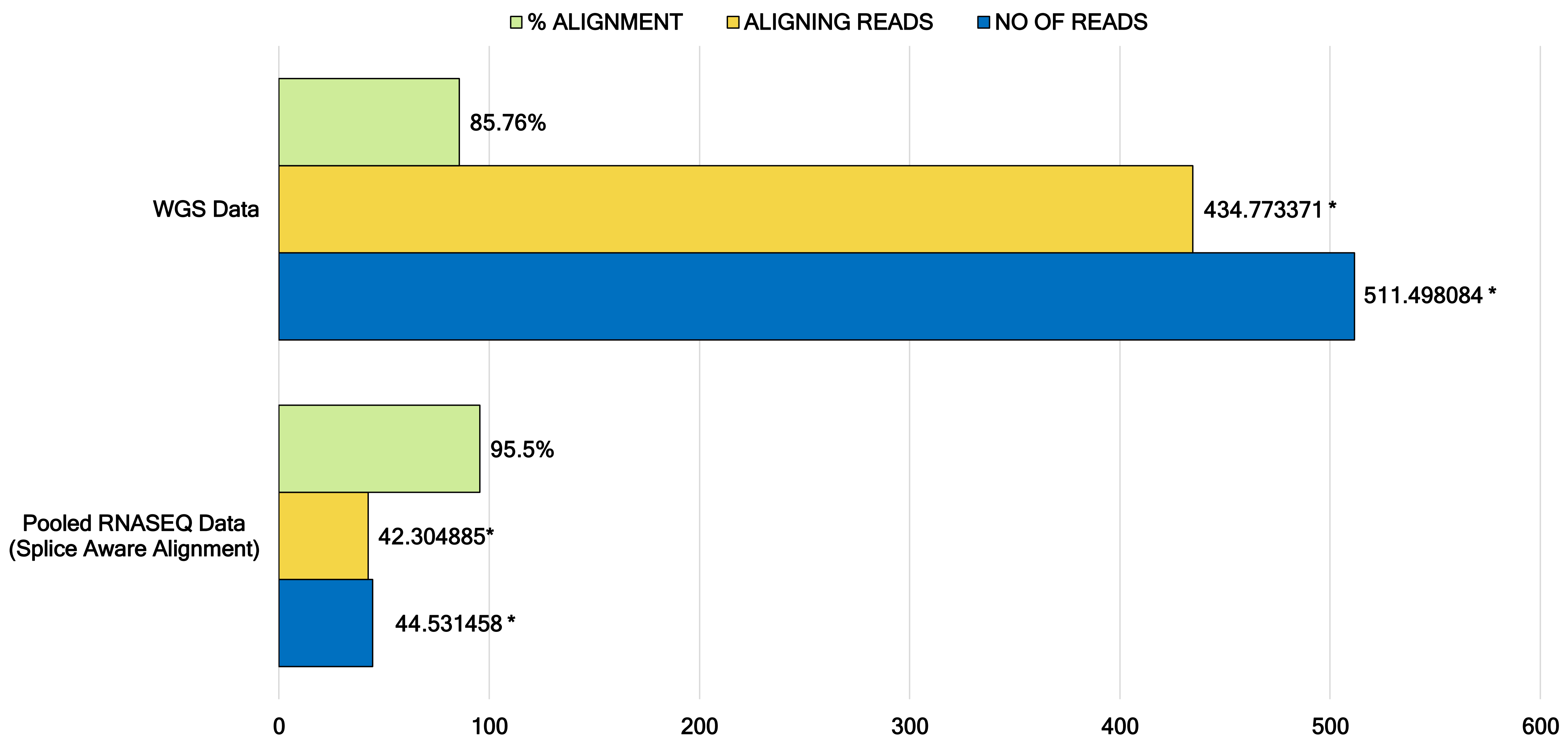

Figure S5. Final genome assembly validation with whole genome sequence (WGS) and pooled RNA-Seq data of *C. zastrowi sillemi.* '*' denotes reads in Million
